# Best Practice: CT‐guided Adaptive Radiotherapy Reference Planning on the Ethos Platform

**DOI:** 10.1002/acm2.70625

**Published:** 2026-05-14

**Authors:** Xenia Ray, Joseph Harms, Alex Price, Mu‐Han Lin, Dennis N. Stanley

**Affiliations:** ^1^ Department of Radiation Medicine and Applied Sciences Moores Cancer Center University of California San Diego San Diego California USA; ^2^ Department of Radiation Oncology Washington University School of Medicine St Louis Missouri USA; ^3^ Department of Radiation Oncology University Hospitals Seidman Cancer Center Case Western Reserve University School of Medicine Cleveland Ohio USA; ^4^ Department of Radiation Oncology The University of Texas Southwestern Medical Center Dallas Texas USA; ^5^ Department of Radiation Oncology The University of Alabama at Birmingham Birmingham Alabama USA

**Keywords:** CT‐guided ART, online adaptive radiotherapy, treatment planning

## Abstract

Online adaptive radiotherapy (ART) enables treatment plan modifications based on the patient's anatomy at each fraction, addressing limitations of conventional radiotherapy that relies on a static pre‐treatment plan. CT‐guided ART (CTgART), as implemented on the Varian Ethos platform, has gained increased clinical adoption over the past several years; however, its success is highly dependent on reference plan design due to limited on‐table planning flexibility. As a result, clinicians creating and reviewing reference plans will greatly benefit from both understanding how upstream planning decisions affect the downstream adaptive process and proactively incorporating strategies that improve optimization robustness and efficiency on‐treatment. We describe a planning framework that emphasizes integration of standard planning while incorporating the temporal workflow considerations. We term this new planning paradigm: Thinking in 4D. In this article, we provide practical recommendations and strategies, gained from high‐volume clinical experience adapting hundreds of cases across multiple institutions, to guide the design of robust Ethos reference planning templates. These recommendations are based on accumulated clinical experience and are intended to complement, rather than replace, formal quantitative validation studies. These principles aim to improve adaptive plan quality, efficiency, and safety, while also supporting implementation, training, and routine plan checks for teams transitioning to planning in 4D.

## 1 BACKGROUND AND OVERVIEW

The clinical emergence of online adaptive radiotherapy (ART) has made it possible to address challenges associated with conventional radiotherapy (RT) treatments. Conventional RT relies on a static treatment plan derived from a single pre‐treatment CT scan. As treatment progresses, changes in anatomy, such as tumor shrinkage, organ motion, or shifts in relative positioning, can lead to deviations between the planned and delivered dose. These differences may result in underdosing the target or overdosing nearby organs‐at‐risk (OARs), particularly in cases with significant inter‐fraction variability. ART offers plan adaptation based on the patient's anatomy of the day, supporting more precise delivery and evaluation of dose. Although the impact of ART on patient outcomes is still being investigated, early experience suggests it may improve plan consistency,^1^, ^2^, ^3^ support dose escalation when appropriate, and better protect normal tissues.^2^, ^4^, ^5^, ^6^, ^7^, ^8^

CT‐guided Adaptive Radiotherapy (CTgART) leverages a cone‐beam CT (CBCT) acquired at the time of treatment to perform on‐table plan adaptation. Compared to magnetic resonance‐guided adaptive radiotherapy (MRgART), which offers excellent soft‐tissue contrast but is limited by cost, infrastructure complexity, and patient MR contraindications, CTgART uses widely available imaging technology that provides sufficient soft tissue contrast and anatomical delineation for many disease sites. As a result, CTgART has become a practical and scalable approach to implementing online ART across a wide range of clinical settings. Since its release in 2019, the Varian Ethos Treatment Planning and Management System (Ethos TPMS) has been adopted in both academic and community‐based practices.^9^, ^10^ Clinical experience has shown that reoptimized, adapted plans are selected for delivery in most adaptive sessions.^11^ Despite growing use, reference planning decisions introduce unique complexities that directly affect the daily adaptive plan quality and efficiency of the on‐treatment process. With the Ethos TPMS, this connection between the reference plan and on‐treatment process is further entangled because the workflow is designed so that adaptors only adjust contours on‐treatment. Unlike current MRgRT systems, all other plan parameters (field arrangement, optimization objectives, normalization, etc.) cannot be changed at an adaptive fraction after the reference plan has been approved. Thus, success with Ethos‐based CTgART depends on a working knowledge of its automation tools, segmentation behavior, prioritization system, planning intent logic, and an understanding of how on‐treatment anatomy changes may affect adaptive plan creation. This manuscript aims to serve as a practical guide for creating robust reference plans that support safe, efficient, and clinically effective adaptation on the Ethos platform.

## 2 CURRENT KNOWLEDGE AND EVIDENCE

### 2.1 Ethos algorithms that enable online adaptation

The Ethos Treatment Planning and Management System (TPMS v3.0MR1, including Treatment Planning System versions v1.0 ‐ v2.1) incorporates several key components designed to support consistent and efficient planning for daily adaptive radiotherapy. These include, but are not limited to, the Intelligent Optimization Engine (IOE), a convolutional neural network (CNN)‐based automated contouring system, and the AcurosXB dose calculation algorithm. Understanding how these tools operate, individually and in combination, is essential for developing robust reference plans that translate into robust adaptive plans. This section outlines the core back‐end planning mechanics and automation strategies within Ethos TPMS that enable CTgART delivery.

#### 2.1.1 Intelligent optimization engine (IOE)

The Intelligent Optimization Engine (IOE v2) is the central planning algorithm in the Ethos TPMS. Full details for its internal logic can be read in the manual^12^ and are described briefly here to highlight key behaviors. The IOE transforms a set of user‐specified clinical goals into an optimization strategy without requiring manual adjustment of objectives or weights by the planner. Clinical goals are categorized by priority levels: P1 (highest) through P4 (lowest), with an optional “report only” category used for metrics that should be tracked without influencing optimization.

Each clinical goal results in one or more internal optimization objectives. The IOE generates and evaluates these objectives iteratively, dynamically adjusting weights based on goal feasibility and priority position. If goals are in direct conflict, such as Target V98% ≥ 35 Gy and an overlapping OAR D0.03cc ≤ 32 Gy, then the IOE will crop whichever structure has the lower priority goal and optimize on only the cropped structure. Each clinical goal may also have a variance assigned, this acts as an upper acceptable limit while the goal is the desired threshold. In addition to user‐specified goals, the IOE will generate additional objectives for targets and OARs to achieve general aspects of plan quality: high target dose conformality, minimizing dose outside of targets, minimizing hotspots, and improving OAR dosimetry. For instance, for targets without a minimum dose goal, the IOE adds up to 3 helper objectives for the lower goal to achieve a tighter shoulder on the DVH curve. For any structure classified as an organ that does not have a mean dose goal, the IOE will generate a mean dose objective and assign it the lowest priority (bottom of P4). The IOE also automatically creates helper structures, such as dose falloff rings, with optimization objectives. These are not user‐editable but are applied internally to promote conformity and influence the shape of dose distributions. Similarly, the IOE uses a Normal Tissue Objective (NTO) to reduce low‐dose spill to non‐target tissue and a monitor unit (MU) constraint to discourage excessive modulation. These features are embedded in the optimizer and require no user input.

All IOE‐created objectives give points to the utility function, which it then monitors to adjust objective weights and objectives. Unlike most optimization algorithms used in treatment planning systems, the IOE continues to search the optimization space to reduce dose for any dose metric, even if that metric has satisfied the specified constraint. This is especially advantageous during an adaptive session, where the IOE begins the reoptimization using the same defined goal hierarchy as the reference plan, but can exploit favorable anatomy to improve dosimetry beyond the reference plan by adjusting the objectives.

#### 2.1.2 Dose preview

Dose Preview provides an interactive assessment of the feasibility of defined clinical goals using a simplified 9 field IMRT beam geometry and a fast dose estimation algorithm. This early preview allows the planner to identify conflicts and unrealistic dose constraints and enables adjustment of relative goal priorities to improve plan quality. When the trade‐offs and estimated plan quality satisfy the planner, they approve the finalized ranked order of clinical goals with variances, and the plan proceeds to full optimization. The fully optimized and calculated dose may or may not match what was predicted in dose preview, depending on the complexity of the plan and the similarity between the field arrangement used for planning and that used in dose preview.

#### 2.1.3 High‐fidelity mode

High‐Fidelity Mode is a planning option designed for SBRT that applies a finer user‐specified dose calculation grid (down to 1.25 mm) and disables the internal dose smoothing filters. High‐fidelity mode also intrinsically adds control rings to the optimization process for conformity and steep dose gradients, and increases the allowable maximum dose limit within the target. Additionally, in recent studies, high‐fidelity mode has also been associated with faster optimization times for SBRT treatment plans.^13^, ^14^, ^15^

#### 2.1.4 Automated segmentation and structure management

In the Ethos TPS, all structures are classified as either targets or organs and assigned a meaning. Meanings can be specific (e.g., gross tumor volume for a target or heart for an organ) or non‐specific (e.g., generic healthy tissue as an organ). The classification and choice of meaning will directly determine at what step of the online process the structure is visible, how the structure is auto‐drawn on treatment, and what options are available for editing. Both targets and organs can be derived by a specific algorithm (e.g., PTV = GTV+5 mm or BowelPRV = Bowel+5 mm) if defined by the user during planning, and if so, will be derived using the same algorithm at each treatment fraction. All non‐derived structures can be manually edited after generation, while derived structures cannot be. On‐treatment, there is no option to add a target or organ structure that did not exist in the original intent.

**Targets:** At treatment, non‐derived targets are segmented using structure‐guided deformable registration based on the influencers. Influencers are organs near or overlapping with the target, which are expected to impact the target shape or position. These are pre‐specified by the software based on the disease site(e.g., if the site is specified as Cervix, the uterus, rectum, and bladder will be the influencers). If influencers are omitted or mislabeled, target propagation may fail or result in poor geometric accuracy.^8^, ^12^ Further details are available in the manual.^12^

The deformed contour can be manually overridden for Targets and replaced with a copy of the target structure from the Reference plan; this is called Rigid Propagation. Both deformed and propagated targets can then be manually edited further as needed. Planners should ensure the appropriate influencers are included and contoured in the structure set if they do not intend to use rigid propagation. This may mean contouring additional structures, for example, the uterus for Cervical cancer patients.

**Organs:** Over 70 organs are auto‐segmented by a convolutional neural network (CNN) based on their assigned meaning during reference planning and on‐treatment adaptive planning, and can then be further edited. During adaptive sessions, any organ without a corresponding CNN, including organs assigned the meaning Generic Healthy Tissue, is propagated from the planning CT to the daily CBCT via a generic deformable image registration. The rigid propagation feature is not available for organs.

#### 2.1.5 Templates

**FIGURE 1:**
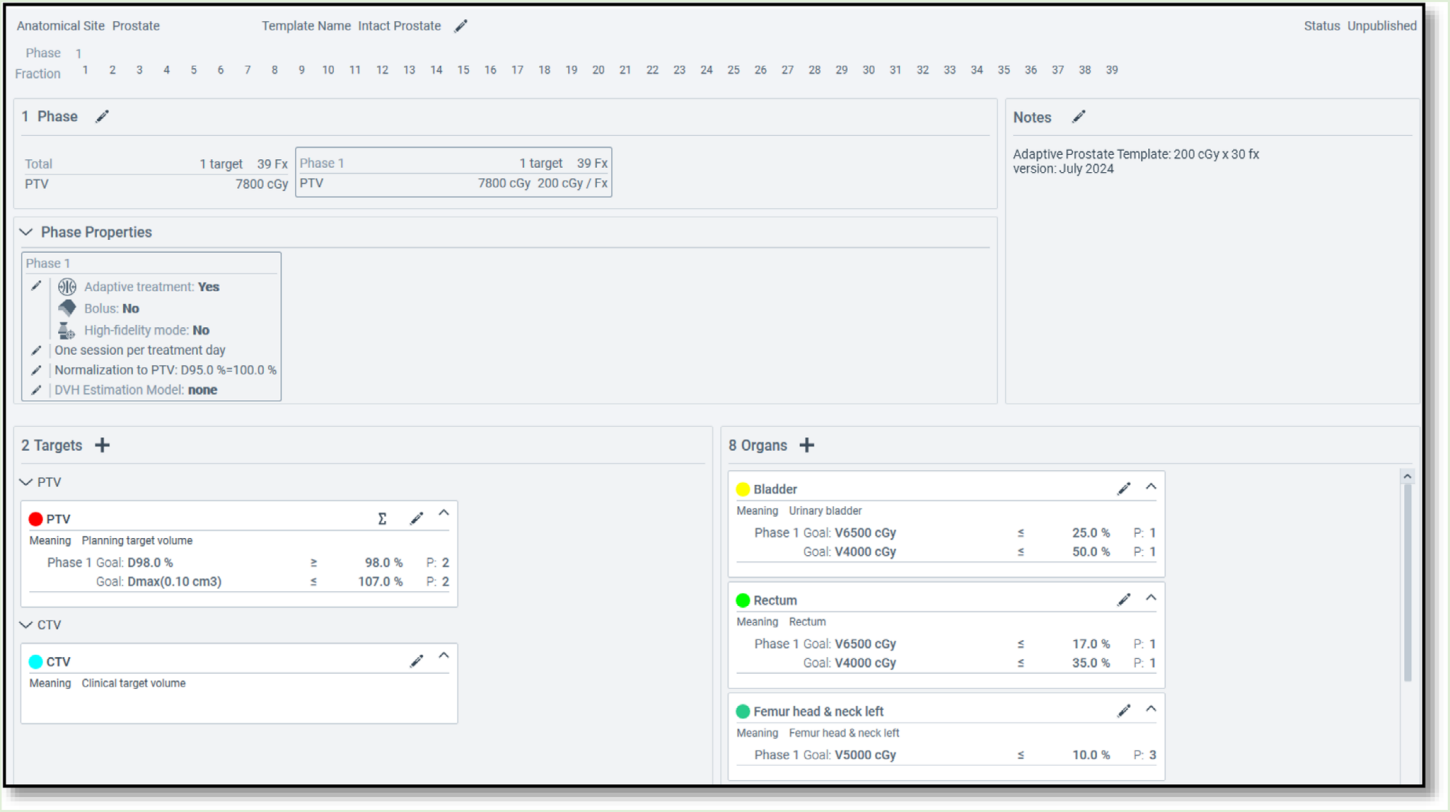
Simple example of an Ethos Planning Template including prescription, structure, and optimization details.

Ethos planning is designed to be template‐based (Figure 1). A plan intent created for a specific patient can be saved as a template and used for later patients of the same disease site and prescription. Alternatively, templates can be designed directly in the Template Management workspace. In either case, templates include the prescription (dose, fractionation, frequency, whether plans will be adaptive, use of bolus, and normalization), structure details (names, meanings, colors, derivations), and guidelines for optimization and plan evaluation (prioritized dose goals, assigned DVH estimation model if used, and use of high‐fidelity mode). As a result, careful design of these templates can standardize treatment planning, improving safety and efficiency.^16^, ^17^ Templates are in .xml format and can be edited outside of Ethos to generate similar clinical intents across multiple treatment sites.

### 2.2 Building a strong reference plan by thinking in 4D

In addition to all standard planning considerations, reference planning for a daily adaptive patient on Ethos requires considering how each choice will affect the on‐treatment efficiency and resulting adaptive plan quality. Because planners must thus consider the effects of each planning choice on the plans generated in the future during adaptive sessions, we term this Thinking in 4D, where the dimensions are 3D dose and time. Thinking in 4D requires an understanding of the algorithms and workspaces described in the previous section to develop a robust treatment planning schema. In this section, we describe each step of the planning process and the unique considerations/recommendations specifically for CTgART on the Ethos. Many of these considerations can be directly built into Ethos Planning Templates to ensure consistency across all planners and improve standard adaptive plan quality. Additional general adaptive planning guidance can be found in the literature, particularly Table 4 of Chetty et al., and is outside the scope of this review.^18^

#### 2.2.1 Simulation considerations

**Immobilization**: Adaptive patients will be on the treatment couch for an extended period of 20–100 min, and on Ethos, no interim imaging can be acquired.^11^, ^19^, ^20^, ^21^ A verification CBCT image can be taken immediately prior to treating the adaptive plan and used to apply 3‐dimensional couch shifts. However, contours cannot be modified on this image, and the plan cannot be reoptimized if internal anatomy has changed during the fraction without restarting the entire session and acquiring a third CBCT. Thus, immobilization devices should be designed at the time of simulation to ensure the patient can remain still for the entire appointment, and considerations of comfort in the immobilization devices should be paramount. On the other hand, inter‐fraction positioning reproducibility is a lesser concern if the plan will be adapted daily, so in certain cases, less immobilization may be appropriate. Some examples of immobilization modifications from conventional RT patient setup may include:

- Including a padded mat on top of the couch for patient comfort
- Modifying vac‐loc bags to position arms above and in front of the patient's head instead of splayed laterally
- Preferring adjustable immobilization devices such as Vac‐Lok bags over fixed devices such as alpha cradles to allow for day‐to‐day adjustments based on patient comfort
- Allowing one or both arms down when treating in the abdomen and using contour‐based goals to prevent dose entering these areas

**Additional Patient Setup Considerations**: In our clinical experience, we have encountered cases where the AI‐based contouring for the body during an adaptive session failed to detect certain bolus materials, such as wet gauze, because of low HU. Similarly, it did not include immobilization devices such as the body frame or breast board. As these omissions can impact the accuracy of online dose calculations, and because the body contour is not editable on the ethos system, the visibility of these devices should be checked during initial program commissioning and adjustments made to simulation protocols if necessary. For instance, users may elect to use water density equivalent bolus sheets in place of wet bolus or replace immobilization frames with those that are radiation transparent.

**Scan Parameters**: On Ethos, the daily CBCT used for planning is acquired with a 2 mm slice thickness. Using the same slice thickness for the CT simulation allows the adapter to more easily compare the daily contours to the reference contours. For instance, they can observe that the reference contours encompass five slices from a specific anatomical landmark and confirm that the daily contours encompass the same range.

**Patient Assessment**: The patient should be assessed for appropriateness as a candidate for adaptive therapy. As an initial filter, their ability and willingness to remain still during the estimated entire treatment appointment duration should be evaluated. A second important criteria is evaluating their likelihood of compliance with specific pre‐treatment preparation instructions (e.g., bladder fill, medications to minimize gas) to reduce intrafraction anatomical changes. Another very practical aspect of compliance can be timely arrival, as delays in adaptive radiotherapy (ART) slots are difficult to recover from in busy clinics. Some patients may not be candidates for daily adapting due to these requirements

**Image‐Only Appointment on Ethos**: To verify that the CBCT image quality at treatment will be adequate for daily adaptive planning, an image‐only appointment can be scheduled on Ethos on the same day as the patient's CT simulation. These appointments are generally not necessary for every adaptive patient, but can provide substantial information and increase confidence levels prior to starting new disease sites or for cases where metal, gas, or internal motion is likely to cause artifacts that would hinder a clinician's ability to confidently contour the target or critical normal tissues. The level of uncertainty in target and OAR delineation gathered from this appointment, combined with the physician's goals for adapting can inform the choice of margins and design of derived structures in the plan. This also allows the planning team to test the AI contouring algorithms on the patient's specific anatomy with the image quality they will see on treatment. Accurate auto‐contouring can promote confidence in the use of specific structures for optimization (e.g., Duodenum), while any observed inaccuracies can be used to inform strategies for generating derived structures for optimization (e.g., Ring structures or OAR Planning Risk Volumes). Lastly, if the clinic has access to an adaptive test environment (e.g., Emulator), the CBCT can be used to perform a dry‐run of the entire adaptive process prior to treatment and ensure all team members are confident in their roles and the tools available for contouring and plan evaluation. This latter step is primarily beneficial when beginning a program, training new team members, or adding a new disease site to the adaptive program.

**Imaging Parameters on Ethos**: Compared to equivalent non‐adaptive treatments, adjusting default CBCT imaging parameters to increase image quality at the expense of imaging dose may be acceptable to decrease uncertainties in localization and contouring during the adaptive process. Thus, therapists and physicists should know when and how to adjust default kVp and mAs to improve image quality. The field‐of‐view should be adjusted to as small as reasonably achievable to optimize trade‐offs between spatial resolution, image noise, and contrast‐to‐noise ratio to improve contouring accuracy. Lastly, consideration should be given to strategies that improve image quality by minimizing intrafraction target motion (e.g., breath‐holds or abdominal compression). A recent study indicated that even allowing a patient's internal anatomy to settle prior to imaging was linked with decreased bowel gas artifact, which could improve target visualization.^22^

#### 2.2.2 Choice of treatment intent

In the Ethos TPS, the first choice the planner must make that will affect the on‐treatment process is the choice of treatment intent. The TPS is set up such that each body site (Head, Thorax, Abdomen, Pelvis, etc) has disease‐site specific intent sites. For example, in the abdomen, the user may choose from subsites such as pancreas, adrenal, or liver. Each body site also includes an option for a generic “other” (e.g., “other abdomen”). In general, for the best results on treatment, the planner should select the site best matching the anatomy, for example, the prostate for a Prostate SBRT treatment plan. However, only one intent of each type can be active at a given time, so for patients who may be receiving separate treatments concurrently in the same disease site (i.e., two liver metastases treated with separate isocenters), the generic “other” intent can be used for the second plan. When the disease being treated does not match any of the predefined disease sites, “other” is also a good default option, though extra care should be paid to whether the default contour list includes appropriate influencers and AI‐drawn contours. Lastly, Ethos treatment planning templates are associated with intents, so in cases where a planning template may be applicable to multiple disease sites within one body site, adding the template with an assigned intent of “other” can be helpful.

#### 2.2.3 Structure set considerations

As mentioned above, all structures are classified as either Targets or OARs and assigned a meaning as well as a name. The classification and choice of meaning will directly determine at what step of the online process the structure is visible, how the structure is auto‐drawn on treatment, and what options are available for editing.

**Auto‐segmentation Evaluation**: Over 70 OARs have independent CNN‐based auto‐segmentation models within the Ethos TPS. To estimate the performance of the on‐treatment OAR CNN‐based auto‐contours, planners should at a minimum run the Ethos TPS auto‐contour on the planning CT during reference planning. For new adaptive disease sites, the likely performance on CBCTs can also be evaluated using the Ethos TPS auto‐contour on CBCTs from previous patients treated in similar anatomical regions. Every structure that will be important for plan optimization or plan evaluation should be evaluated to determine if edits to the CNN‐based auto‐contoured structure would be necessary but achievable within an adaptive appointment or too involved to be feasible. If the latter is true, the structure may need to be unmatched from its meaning to prevent the auto‐contour from being run at treatment, and/or an alternative optimization or evaluation‐based structure should be created to serve the purpose of the initial structure.

Evaluating systematic differences between the Ethos auto‐segmentation algorithm and institutional contouring practices, or separate institutional auto‐segmentation models is especially important for thinking in 4D. For patients receiving adaptive therapy over a conventionally fractionated RT course, these systematic differences would need to be corrected 20–30 times. Depending on adaptive coverage models, it is likely that multiple adaptors will edit the contours. Therefore, leaving systematic differences in the structure segmentations could lead to variable plan quality.

Some examples of these differences noted from our clinical practices are listed below:

- The CNN was observed to perform poorly for the Left and Right Kidney, thus the authors linked these OARs to the Generic Healthy Tissue meaning. On treatment, these structures are auto‐contoured using deformable registration and require significantly less edits than correcting the CNN‐based contours.
- For patients with ascites, the liver was set with a meaning of Generic Healthy Tissue instead of liver because the deformation performed better than the CNN auto‐contour.
- The CNN for BowelLoops was observed to work very well, but occasionally included some of the pelvic vessels, which were included in the target CTVs when treating pelvic nodes. A secondary OAR was set up using the derivation: BowelFinal = BowelAutoContour‐CTV to reduce the number of manual edits needed.
- Distinguishing large bowel, small bowel, duodenum, and stomach OAR boundaries was a challenge for a specific patient due to image quality. Instead of manually editing each of these, a BowelSpace‐CTV structure was used with the strictest DVH criteria from any of these potential OARs enforced as an optimization goal.
- A large cyst near the bladder was erroneously included in the bladder autocontour. To alleviate this, the following were added: BladderAutoSeg with meaning Bladder, Cyst with meaning Generic Healthy Tissue, and Bladder with meaning Generic Health Tissue derived as BladderAutoSeg cropped away from Cyst.
- To increase efficiency on‐treatment, a Region of Interest (ROI) structure is used, and OARs are only edited within this structure. To ensure the ROI structure does not get deformed, it is labeled as a Target during initial planning and rigidly propagated on‐treatment.

**Structure Naming and Color**: Target and OAR structures should be clearly labeled to indicate what anatomy they should include, whether they will have further derivations applied, and if (in the case of targets) rigid propagation should be used. A standard nomenclature for on‐treatment adaptive, regardless of platform, has been jointly recommended by TG‐395 and TG‐263U1 and at the time of writing are under review. When published, clinics should review these resources for adopting a standard nomenclature.

Colors of structures should also be standardized to facilitate coverage at the machine. In addition to having set colors used for targets that need editing and standard colors for critical OARs, it can be beneficial to use a neutral grey color for all derived optimization structures or similar that do not need to be edited or evaluated during a session.

**Individual Targets**: In Ethos TPMS, it is helpful to label discrete targets separately rather than combining them into a single volume so they can be propagated independently. If multiple lesions or regions of disease are included in one structure, the system may interpolate between them during adaptive deformation or edits on treatment, creating non‐physical bridging or incorrect shapes. If targets are meant to remain separate, for example, multiple lymph nodes within one GTV_High, a best practice is to contour each target volume individually, numbered in a systematic way (e.g., superior to inferior) and follow a certain color code (e.g., rainbow order) to ensure efficiency of alignment and avoid confusion when aligning upwards of 3 individual targets. These can then be logically combined using a derived structure (e.g., union of GTV1‐10 to form a planning target volume) for use in optimization. This approach improves structure tracking, preserves anatomical realism during adaptation, and supports more consistent plan quality across fractions. Finally, if rigidly propagating targets, one can add a structure contour for implanted fiducials or in vivo anatomic landmarks to aid in target localization. For example, in the setting of the pancreas, vessels are delineated as “NS_AlignVessel,” and are encapsulated within the full GTV so that when the GTV is rigidly propagated, the system also propagates NS_AlignVessel, and the vessels can be used to help align the pancreas GTV.

**Target Derivations**: Careful consideration should be given to the derivations used for targets. For instance, for prostate, clinics may opt to rigidly propagate or use the deformed CTV_Prostate and then use a derived PTV = CTV_Prostate +margins. Alternatively, they can create the PTV by subtracting normal anatomy, for example, PTV = (CTV_Prostate—Bladder—Rectum—Sigmoid)+margins to reduce the number of edits they need to make to the CTV_Prostate. Derivations can be more elaborate, including multi‐steps to allow for subtraction of PRVs or asymmetric margins in the directions of different priority OARs. The best choice for each disease site and prescription regimen will depend on the goals of the adaptive treatment (safe dose escalation vs ensuring standard target coverage vs maximizing OAR sparing), image quality for the disease area, and efficiency of using deformed vs propagated vs auto‐contoured structures.

To help with plan evaluation, adding PTV_Opt structures (equal to PTV structures with OARs or high dose PTVs subtracted with or without additional margin) with threshold tumor coverage metrics are also useful to ensure that, regardless of scenario specifics, the plan evaluators can always tell if the final plan has covered the area where the prescription dose should be.

**The Body**: Decreasing the overall volume of the body can significantly decrease the optimization time, especially for VMAT plans or plans using high‐fidelity mode. In these instances, truncating the body or simulation CT scan in the superior and inferior directions should be considered, though enough additional tissue should be kept to accurately represent scatter to the relevant tissues.

#### 2.2.4 Design and prioritization of clinical goals

The algorithms used by the Ethos Intelligent Optimization Engine (IOE) to optimize a treatment plan have been described in detail in the literature and above.^23^ A key feature of the IOE is its “meet and beat” optimization behavior. During an adaptive session, this same list of prioritized goals from the reference plan is used to reoptimize the plan. The optimizer converts goals to objectives and pushes on them beyond what has been listed as the goal, allowing for some added robustness to daily, reasonable anatomical changes. Additionally, it is important to note that, unlike traditional planning, the design of the prioritized goals must balance between plan quality/safety and planning efficiency. Increasing the number of structures with goals increases the time required for contouring, as each should be evaluated at each treatment, and adding significantly more goals to the list can increase the optimization time. We recommend the following general planning principles for consideration.

**Adding Goals**: Any clinical goal that would be checked to evaluate the plan's safety and quality should be included. However, to help with optimization efficiency, many of these can have their category assigned as ‘Report Only’ for both targets and OARs. Goals in category 1–4 should only be added to structures that will be reviewed (and edited if necessary) on‐treatment. When adding goals to these categories, consideration should be given to how that structure will be drawn on treatment and if editing it will be needed and/or feasible within the adaptive window. For example, large volumetric structures may be unreliably evaluated since it may be time‐consuming or not possible to have the full volumetric volume within the scan. Goals to achieve the required dose fall off effect can be strategically added to derived structures instead of the large volumetric OARs, such as cropped OARs, OAR PRVs, Rings, etc., depending on the context.

**FIGURE 2:**
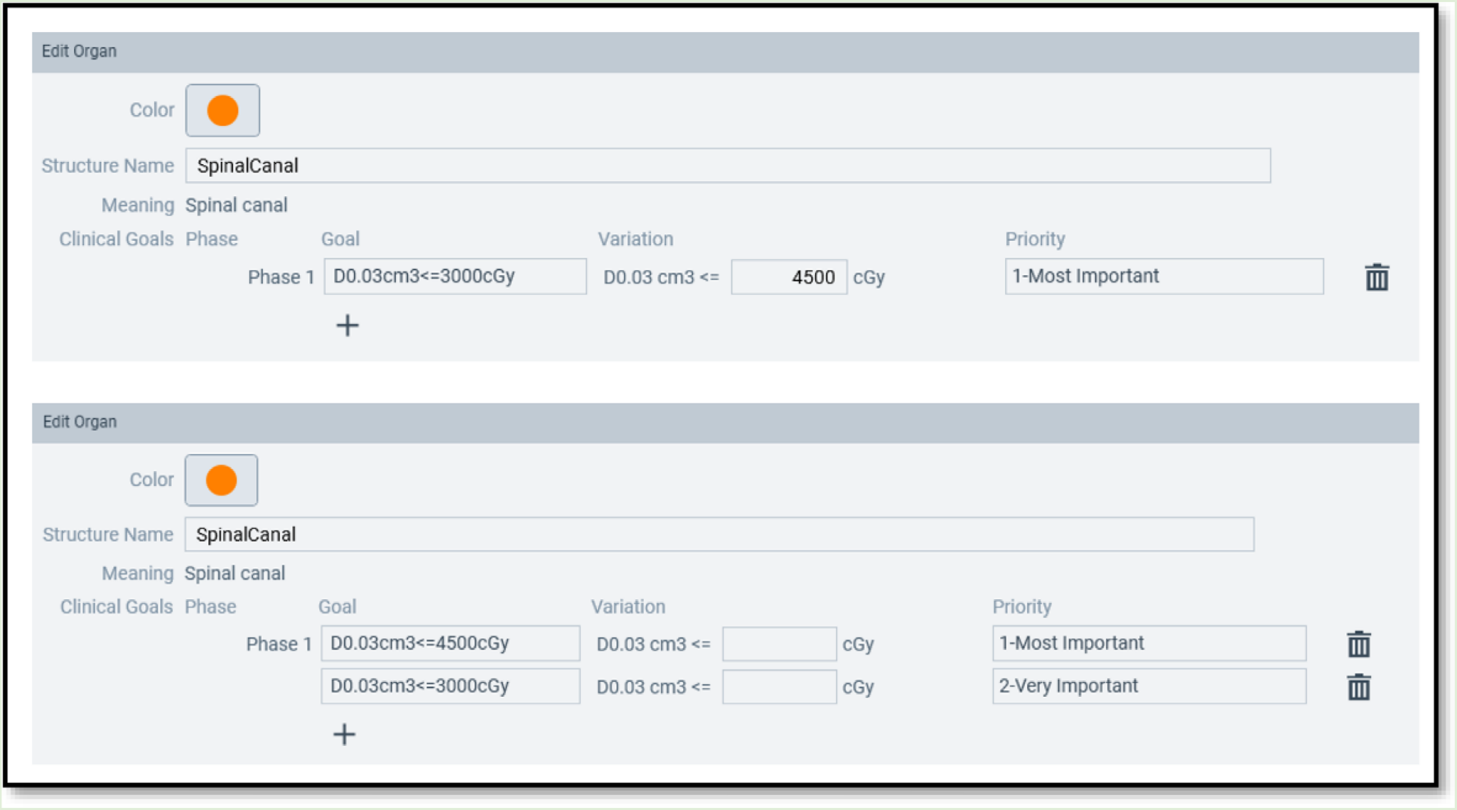
Example of two ways to encode soft and hard organ‐at‐risk limits in an Ethos template. Top: a single spinal canal goal is entered with an aspirational objective of D0.03 cm^3^ ≤ 3000 cGy and a variation of 4500 cGy, allowing the optimizer to push toward the lower dose while preserving 4500 cGy as the upper acceptable limit. Bottom: the same planning intent is represented using two separate goals: a hard limit of D0.03 cm^3^ ≤ 4500 cGy assigned higher priority, and a softer planning objective of D0.03 cm^3^ ≤ 3000 cGy assigned lower priority. This illustrates two practical strategies for handling goal “meet‐and‐beat” behavior in Ethos.

**Adding Variations**: For each DVH metric used for planning, the variation in Ethos can be set to define the clear upper limit, while the goal value can be set to push the plan a bit further to within preferred limits when possible. Planners can leverage this behavior by defining realistic but aspirational goal values, paired with clear variance thresholds, to guide the IOE toward high‐quality outcomes that respect clinical boundaries, Figure 2 (Top). Alternatively, the dose metric can be split into two goals, one with the hard limit assigned a high priority and one with the softer reach limit assigned a lower priority, Figure 2 (Bottom). This approach has the advantage of giving the planner more control over how each limit is used within the optimizer and prevents the IOE from overly pushing for the soft limit at the expense of other goals when it is not feasible. In this case, the softer limit may flag as red on treatment. Adapters can be trained to understand that goals not in priority 1 are not hard stops on treatment, or the hard limit can be added as a variation on the soft goal in priority 2.

Overall, goals should reflect the expected trade‐offs in coverage and sparing, and their priority ordering should be based on clinical evaluation. One important note, while safety‐based constraints such as those from QUANTEC are critical for defining the bounds of acceptability, we have found that applying just these values as goals to treatment plans can lead to relatively low‐quality plans.

**Assigning Priorities**: General prioritization of goals should match clinical preferences. Thus, if it would never be acceptable to exceed a particular threshold on an OAR even if it means undercovering the PTV, then that goal should be the first goal in Category 1. For example, in our practice for SBRT within the abdomen, we employ an isotoxicity approach and typically reserve P1 only for gastrointestinal dose constraints,^24^ and put all GTV and PTV objectives and goals within P2 to help ensure OAR tolerance doses are not exceeded. However, caution should be employed when assigning OAR constraints to a higher priority than PTV coverage because of the IOE's meet‐and‐beat strategy. As an example, consider a pancreatic SBRT plan, with PTV prescription of 50 Gy. If constraints are defined as Duodenum D0.5cc < 3300 cGy in P1 and PTV/GTV coverages are only listed in P2, the IOE will treat the PTV as being cropped from inside the duodenum, and it will reduce duodenum D0.5cc as low as is achievable, possibly leading to very low doses within the target. If, however, the duodenum constraint was defined as V3300 cGy < 0.5 cc, the IOE will only optimize on voxels within the duodenum receiving > 3300 cGy. Defining this constraint differently can thus impact the minimum dose to the PTV or GTV.

**Including RapidPlan Objectives**: RapidPlan models designed in Eclipse can be used by Ethos to create line objectives for any matching OARs. These line objectives are automatically assigned a priority at the bottom of Category 2.^5^, ^6^, ^11^ During an adaptive session, the line objective will be recalculated based on the anatomy of the day, allowing the optimizer a realistic estimate of what is possible and helping the goal template evolve with changing anatomy.^25^, ^26^ This is of particular use in cases such as a large shrinking target to help increase the sparing of surrounding normal tissues.

#### 2.2.5 Field configurations and additional considerations

**VMAT versus IMRT**: For online adaptive sessions, IMRT plans will optimize and calculate dose in 2–8 min while VMAT plans can take between 4–12 min depending on the volume of the body, complexity of the prioritized clinical goal list, and dose grid resolution. However, VMAT plans will require fewer MU and deliver substantially faster for the same dose. A recent study performed a direct comparison using the latest Ethos optimization algorithms and found that for 15 lung SABR patients, IMRT plans completed in less than 90 s while high fidelity mode VMAT plans required 269 ± 62 s.^14^ In the same study, the MU for the IMRT plans ranged from averages of 3225–4508 MU depending on beam arrangement and optimization goals, while the VMAT plans had a lower average of 3142 MU.^14^ In practice, the authors typically use VMAT beam arrangements for SBRT plans (because the dose to deliver is high and the increasing MU with IMRT becomes a significant time increase) and IMRT beam arrangements for conventionally fractionated plans (where the decreased optimization time cancels out the increased delivery time and can thus reduce the amount of time physicians are needed at the machine). For breath‐hold treatments, the time the patient will be in a breath‐hold state will be substantially less with VMAT, which will lead to better patient compliance.^27^

**Normalization**: Plan normalization must be defined as part of the initial planning intent, and the same normalization is used at each adaptive fraction. Options include not normalizing, normalizing a target to a specific value (PTV V100% = 95%), or normalizing to meet all priority 1 OAR goals (isotoxicity approach). Because of the unique behavior of the IOE, and because the normalization strategy cannot be changed on treatment, careful thought should be given to this choice. If there is a specific OAR metric that must be met, then normalizing to a PTV might erase the delicate balance between target coverage and normal tissue sparing achieved by the optimizer. While in some circumstances, normalizing to meet all priority 1 OAR goals can under normalize the plan leading to unnecessarily low target coverage. Observing the behavior of the plan with and without normalization during reference planning can help guide the best decision for on‐treatment. Additionally, on‐treatment review of the final normalization value per fraction can identify potential reasons for adjusting one or more features of the reference plan.

#### 2.2.6 Planning with multiple margins

Daily adaption may allow for margin reduction, particularly in disease sites where standard plans used an ITV approach to account for inter‐fraction motion (e.g., variation in uterus position with bladder fill). However, adapting a plan also introduces the risk that the newly optimized plan at any given fraction may be unsuitable for treatment 25–27. For instance, it could exceed a critical OAR tolerance metric or fail to calculate at all. In these instances, users may prefer to treat what Ethos labels as the ‘scheduled plan’. This is the plan that was initially approved in the Ethos TPS recalculated on the image and contours of the day. However, if margins were substantially reduced to generate this scheduled plan, then it may also be unsuitable for treatment at any given fraction. To circumvent this and improve the likelihood that the scheduled plan is generally acceptable for treatment in cases where the adaptive plan fails, users can make use of the fallback planning workflow on Ethos to create a course where the scheduled plan uses standard non‐adaptive clinical margins and the generated daily adaptive plans use smaller margins specific to the anatomy of the day.

To achieve this, the following steps are recommended:

1. Users create an intent with three PTVs
  - PTV_Standard = CTV + standard non‐adaptive margins
  - PTV_Adaptive = CTV + smaller adaptive margins
  - PTV_Final = PTV_Standard
2. All dose goals for the PTV are assigned only to PTV_Final (or other PTV structures derived from PTV_Final such as PTV_Opt if multiple dose levels).
3. Dose goals are added to the OARs and other optimization structures as normal and a plan is generated that is acceptable for treatment, assuming full dose to PTV_Final = PTV_Standard.
4. The plan that is generated is exported to a local drive as an RP dicom file, ‘Plan_Standard’
5. The intent is revised so that PTV_Final = PTV_Adaptive and re‐optimized to generate a plan that is acceptable for treatment, assuming full dose to PTV_Final = PTV_Adaptive.
6. If needed, dose goals on OARs and other optimization structures are updated to improve plan quality for the smaller PTV. The larger the margin reduction, the more likely it is that this step will be required.
7. The ‘Plan_Standard’ is imported to the existing intent.
8. Upon a single plan import, Ethos automatically creates two plans:
  - Imported and Recalculated Plan: recalculates the plan with fixed Monitor Units, thus preserving dose to PTV_Standard.
  - Imported and Reoptimized Plan: re‐optimizes the plan using the current intent and prioritized dose goals with the imported plan field geometry, and thus optimizes dose to PTV_Adaptive.
9. The physician reviews both of these plans and approves the first one for treatment.
  - This plan becomes the scheduled plan and thus will include the larger dose cloud to cover PTV_Standard during any given adaptive fraction.
  - The second plan should be reviewed but not approved because it is representative of the plan quality and dose trade‐offs users can expect during an adaptive session in the adaptive plan. In particular this plan should be checked to confirm that it gives an appropriate dose to PTV_Adaptive and does not optimize to PTV_Standard

This approach allows for safer implementation of reduced margins in adaptive radiotherapy, as daily review can always treat with the scheduled plan that matches the non‐adaptive standard of care volumes. As a secondary benefit, this approach improves plan interpretation during chart review and billing, as the scheduled plan remains aligned with conventional planning expectations. An alternative approach used at some institutions is to maintain a separate RT Intent containing a non‐adaptive plan with standard margins, allowing clinicians to switch between intents depending on daily plan suitability. Both strategies can provide a clinically effective fallback option, and the optimal choice may depend on institutional workflow preferences and data management considerations.

#### 2.2.7 Pre‐Planning and Treatment Reviews

Considering the specialized nuances listed in section 2.2 above, we have found implementing pre‐planning reviews by a physicist that covers adaptive treatments to be highly beneficial. In a retrospective assessment of adaptive clinical practice, Iqbal et al found that employing a pre‐planning review to ensure contour definitions and derivations and planning templates led to decreased overall planning time because adaptive ‘thinking in 4D’ errors were caught earlier in the planning process.^28^ When planning is complete and doses are approved by physicians, we have also found it essential for safe clinical implementation for the final physics plan‐check to be completed by physicists with experience covering adaptive treatments.^29^ This allows for the final review to go beyond the final plan dose and parameters to include review of the contour derivations, structure colors, prioritized dose goal list, normalization, field arrangement from the perspective of daily adaptive plan robustness, efficiency, and safety.

## 3 BEST‐PRACTICE RECOMMENDATIONS

The best‐practice recommendations in Table 1 below synthesize practical lessons learned from high‐volume adaptive clinical experience and are organized by workflow steps to improve robustness, efficiency, and safety during daily adaptation and routine review.

**TABLE 1:** Practical best‐practice recommendations for Ethos CT‐guided adaptive radiotherapy reference planning, organized by workflow step to improve robustness, efficiency, and safety.

Domain	Decision point	Best‐practice guidance	Why it matters (Robustness, Efficiency, and Safety)	Practical implementation notes
**Patient selection**	Tolerance for time/motion	Select patients who can reliably tolerate session preparation and duration	Prevents intrafraction change and workflow failure	Use screening questions + pre‐scan immobilization assessment
	Patient Compliance	Evaluate patient compliance for preparation instructions and ability to maintain position for extended treatment time	Adaptive sessions require more clinical resources than conventional RT. Missed appointments or delays caused by compliance issues provide greater disruption to clinical workflow	Establish realistic expectations with the patients on appointment length and the importance of timeliness
**Simulation imaging**	Immobilization	Prioritize comfort + stability for prolonged couch time	Reduces intrafraction motion	Pads, reproducible supports, breath‐hold coaching
	Follow adaptive‐specific setup considerations	Design protocols that prioritize reproducible internal anatomy and patient comfort for techniques such as treatments requiring bladder filling and breath‐holds.	Setup conditions for typical radiotherapy treatments may be too strenuous for adaptive sessions	Confirm that the patient can comfortably maintain a full bladder for 30–60 min or reliably perform repeated breath‐holds throughout the treatment session
	Image clarity and target visibility	Acquire an Ethos CBCT prior to planning for patients when artifacts or visualization concerns are expected	Confirms target and OAR visibility prior to treatment planning	Particularly useful for new disease sites, metal implants, or gas‐prone anatomy
**Intent creation and planning templates**	Intent selection	Choose the disease‐site intent that best matches the anatomy being treated	Improves adaptive contour behavior and data mining and analysis.	Use the defined intents specific to the treatment location. Save “Other” intents for nonstandard cases.
	Template design and standardization	Develop standardized planning templates that include prescription, structure definitions, meanings, derivations, and prioritized clinical goals.	Improves planning consistency and reduces variability across planners and adaptive sessions	Maintain templates centrally and apply consistent structure naming, colors, and derivation logic
	Use of “Other” Intents	Use a generic “Other” intent when multiple plans are required within the same body site	Ethos only allows one active disease‐site intent of a given type, and conflicting intents can disrupt adaptive workflows	For example, when treating multiple liver metastases with separate isocenters, assign the second plan to “Other Abdomen”
**Contouring/ structure set**	Structure naming and color conventions	Use TG 263‐compliant naming* and standard color schemes for targets, OARs, and derived structures *Default Ethos TPS names are not TG‐263 compliant and need to be manually modified by the user	Reduces confusion during on‐treatment contour editing and plan review, allows for easier handoffs between adaptors	Use consistent and high contrast visibility colors
	Multi‐target management	Keep spatially distinct targets separate and combine via derived structures for optimization	Prevents deformation artifacts and allows for independent propagation	Number targets systematically and use consistent color coding
	Structure meaning assignment	Assign appropriate structure meanings to control whether organs are auto‐segmented or deformably propagated	Structure meaning determines how contours are generated on treatment and affects editing workload	Bowel should contain only bowel loops and not the bowel space. If systematic inaccuracies occur, adjust structure definitions or use derived structures (e.g., cropped bowel structures) to maintain consistent anatomy for optimization
	Structure availability at treatment	Ensure all targets/OARs needed for optimization/evaluation exist in the intent/template at reference planning and have at least one goal	Goals and structures Cannot be added on‐session	List fiducials or structures to guide propagation/deformation (e.g., stents) as targets
**Optimization & algorithms**	Dose Preview and Goal feasibility and conflicts	Use Dose Preview to evaluate feasibility of clinical goals and detect conflicts prior to full optimization, with particular importance in adaptive workflows where on‐treatment adjustments to planning parameters are limited	Prevents inefficient optimization cycles and unrealistic goal sets	Note that the dose preview is generated using a standard 9‐field beam arrangement; users should consider differences between this configuration and the case‐specific beam arrangement when interpreting the preview and determining the appropriate course of action.
	Technique selection (IMRT vs VMAT)	Evaluate the trade‐offs between optimization speed and delivery efficiency	Impacts total adaptive session time and patient motion tolerance	IMRT often optimizes faster VMAT often delivers faster with fewer MU, which is particularly advantageous for breath‐hold cases
	High‐Fidelity Mode usage	Use for plans sharper dose gradients or higher in‐target hot spots	Produces SBRT‐like normal tissue objective	Plan quality is dependent on goals of treatment and expected dose falloff
	Optimization complexity / Use of support / helper structures	Limit unnecessary structures and objectives in optimization Use additional support structures (e.g., rings, PRVs, cropped OARs) only when they meaningfully guide dose shaping	Excessive helper structures may increase optimization and treatment planning time without improving plan quality	Limit support structures to cases where they provide clear benefit. In many cases, similar optimization behavior can be achieved by adjusting goal priority rankings rather than adding additional structures.
**Clinical goals (Priorities, Variances, Metrics)**	Priority hierarchy	Put non‐negotiable safety constraints above preference metrics	Ensures critical OAR limits are protected and prevents unsafe trade‐offs during optimization	Document the intended priority hierarchy within planning templates and review it during plan evaluation
	Hard vs soft limits	Encode hard clinical limits using the variance value and set the goal value as the patient‐specific or aspirational planning objective	Allows the optimizer to push plan quality while maintaining clear clinical safety thresholds	Use variance to represent the maximum acceptable dose while the goal represents the desired planning target
	Maximum dose constraint formulation	Avoid using absolute Dmax constraints and instead use small‐volume metrics such as D0.1cc or Dmax0.1cc.	Dmax can optimize on a single voxel, which can be unstable and overly sensitive to contouring or dose calculation variations	Use small‐volume maximum dose metrics (e.g., D0.03–0.5 cc depending on organ and prescription) to create more robust and clinically meaningful constraints
	Goal Meet‐and‐beat behavior	Set goals and priorities with the understanding that IOE will continue improving metrics beyond the specified goal when possible	Exploits favorable anatomy during adaptive sessions but can also unintentionally reduce target dose if priorities are poorly defined	Implement dual goals (e.g., hard constraint in higher priority and softer goal in lower priority) Focus optimization on structures that meaningfully influence dose distribution
	Goal list efficiency	Limit P1–P2 goals to structures that will be reviewed/edited daily; use report‐only status to metrics intended primarily for monitoring or evaluation.	Improves on‐couch efficiency, reduces optimizer burden, and cognitive burden on session for adaptors	Avoid high‐priority goals on very large structures like the body
	Fallback option of Daily Adaption	If using reduced margins for adaption, maintain a fallback strategy using standard clinical margins	Ensures a safe treatment option exists if the adaptive plan cannot be generated or fails to meet clinical criteria	Consider maintaining a scheduled plan using standard margins while adaptive plans use reduced margins to account for daily anatomy
**Pre‐planning and plan review**	Normalization strategy	Evaluate the normalization strategy during plan review since it cannot be modified during adaptive sessions	Incorrect normalization can alter the intended balance between target coverage and OAR sparing during daily optimization	Compare plan behavior with and without normalization during reference planning and monitor normalization values during treatment fractions
	Adaptive‐focused pre‐planning review	Implement pre‐planning review by clinicians who cover adaptive sessions	Catches derivation/template/structure issues early and reduces overall planning time	Review intent selection, structure meanings and derivations, influencer strategy, and whether goals rely on editable anatomy
**On‐session considerations**	Escalation and decision rules	Define “treat adapted vs treat scheduled vs restart” triggers before first fraction	Prevents ad hoc decisions under time pressure and improves safety	Use a simple decision tree tied to non‐negotiable metrics and calculation failures
	Clinically meaningful contour edits	Limit contour modifications during adaptive sessions to clinically meaningful edits that impact dose distribution	Excessive or cosmetic edits increase on‐couch time	Focus edits on high‐priority targets and OARs influencing optimization outcomes; avoid unnecessary refinement of structures that do not affect clinical goals or are very far from targets
	Verification CBCT prior to delivery	Acquire a verification CBCT before treatment delivery to confirm patient positioning and internal anatomy stability	Detects intrafraction motion or anatomical change that may have occurred during the adaptive workflow	Apply couch shifts if needed based on verification imaging; if significant anatomy changes are observed, the adaptive session may need to be restarted
	Intrafraction motion monitoring	Use surface imaging when available to monitor patient motion during the adaptive workflow and treatment delivery	Adaptive sessions are longer than conventional treatments, increasing the risk of patient motion	Configure motion thresholds appropriate for the treatment site and use surface imaging to verify patient stability while the plan is being generated and during delivery Re‐CBCT during adaptive sessions if internal motion is expected (i.e. bowel movement or bladder filling)
	Imaging parameters (kVp/mAs/FOV, motion mitigation)	Optimize CBCT image quality for adaptive contouring (accepting higher imaging dose when appropriate)	Contour uncertainty drives plan uncertainty and increases edits/on‐couch time	Train staff on when/how to adjust kVp/mAs; minimize FOV; consider breath‐hold/compression; allow anatomy to settle to reduce bowel gas artifact

## 4 PRACTICAL CONSIDERATIONS

Errors or unexpected system behavior may occur during the initial reference planning or during an on‐treatment session. In this section we outline common error modes and steps for their resolution.

### 4.1 Calculated plan is lower quality than prediction in dose preview

When planning, users may find that the predicted DVH metrics in Dose Preview are not achieved in the fully calculated plan. Some degradation of plan quality is expected with the differences in algorithms and more realistic dose deposition used for the full calculated plan. Additionally, in many instances, the beam geometry used for the calculated plan may substantially differ from the 9 field IMRT arrangement in Dose Preview. To help identify what should be changed in the prioritized list of dose goals, it can be helpful in some instances to review dose normalization (is the plan being significantly normalized up to get coverage or down to spare OARs).

### 4.2 Online adaptive plan exceeds critical plan quality metrics

The adapter may observe that the adapted plan on‐treatment did not achieve the intended compromise between target coverage and normal tissue sparing, and non‐negotiable metrics are exceeded. There is no option to adjust the prioritized list of clinical goals on‐treatment, so if this is observed, adapters have three options: treat the scheduled plan (assuming it meets relevant metrics), cancel the treatment and make adjustments to the intent, then re‐start the treatment, or change the results of the optimization by adjusting the online contours. This latter option can work in instances where an OAR maximum dose (dmax) is exceeded, by either drawing the OAR slightly larger or shrinking the boundary of the target in the direction of the OAR to force a larger separation between the two volumes and ideally move the offending dmax out of the true OAR. Offline changes to the reference plan can then be made to automate this behavior, for instance, by moving OAR clinical goals to an OAR PRV that is prioritized over target coverage or to an optimization structure that has no anatomical tie‐in (e.g., z_CoolDown) that can be drawn on treatment to get the desired dose distribution.

### 4.3 Adaptive plan quality may degrade over time

The initial reference plan prioritization of goals is generally tuned for similar anatomy as was visualized in CT simulation. However, if the patient is on‐treatment for multiple weeks, their targets may shrink, or the relative position of OARs may change. As a result, the adapted plan may meet all metrics but fail to spare the OARs as much as possible in the new anatomy geometry. If observed, the list of goals should be updated to create a more optimal plan. Including report‐only metrics on rings at set distances from the target can help indicate if adapted plans are failing to maintain the expected dose gradient as the target shrinks. Other variations due to relative shifts in positions of OARs can be harder to detect and rely on experienced plan review of the daily adaptive plan. Using RapidPlan line objectives can ensure the OAR clinical goals are dynamic to these changes, but unfortunately these cannot be directly viewed at‐treatment to confirm optimal OAR sparing.

## 5 ADAPTIVE PLAN MAY FAIL TO CALCULATE

This may be the result of a technical glitch, or intentional safe‐guarding behavior. The Ethos TPS calculates several safety checks prior to presenting the plan on‐treatment and will not show a plan that cannot pass these checks. The safety checklist includes normalizing above a set threshold, which is a global parameter set in the TPMS. This can occur if a small voxel or more of target was accidentally drawn outside the treatment field of view. Similarly if the patient setup is offset to the point that the max field opening cannot deliver dose to the treatment area, it will not generate a plan. Lastly, errors can be introduced by AI contouring failures. If the AI cannot contour a structure reasonably accurately in the initial sim, and creates a non‐anatomic structure, it is best practice to turn off the AI contouring for that structure to prevent issues on treatment. Anytime an adaptive plan fails to calculate, the adapter should carefully review the target and high‐priority OAR contours, ensuring their integrity. If no clear issues are spotted, making a small clinically insignificant change to one contour will trigger re‐optimization and can clear errors due to minor technical glitches.

## 6 CONCLUSIONS

Success with CTgART depends on a working knowledge of its automation tools, segmentation behavior, prioritization system, and planning intent logic. We acknowledge that adaptive radiotherapy platforms, including Ethos, are rapidly evolving, and that system behavior, particularly related to contour propagation, auto‐segmentation, and optimization algorithms, may change in future software versions. The recommendations presented here are based on the current system configuration (TPMS v3.0MR1, TPS v2.0) and reflect best practices at the time of writing. Despite this, we anticipate that the underlying principles of robust planning, prioritization strategies, and workflow‐aware design will remain broadly applicable across future system iterations and other CT‐guided adaptive radiotherapy platforms. The practical principles described in this manuscript can be used to design Ethos planning templates that improve adaptive robustness, efficiency, and safety. Additionally, they can form the basis of a plan review checklist specific for adaptive cases during implementation, training, and routine QA to help dosimetrists and physicists as they learn to think and plan in 4D.

## FUNDING INFORMATION

Research reported in this work was supported, in part, by funding from the National Institutes of Health National Cancer Institute award (L30CA284339) to Dennis N. Stanley, Ph.D.

## CONFLICT OF INTEREST STATEMENT

Xenia Ray and Dennis N. Stanley receive consulting fees, payment or honoraria for lectures, presentations, speaker bureaus, manuscript writing, or educational events, and support for attending meetings and/or travel from Varian Medical Systems as Educational Consultants. Alex Price reports honorarium from Radformation, Inc., and stock options from Aitrium not related to this work. The other authors declare that they have no known competing financial interests or personal relationships that could have appeared to influence the work reported in this paper.
